# Antimicrobial Resistance Profiles, Virulence Determinants, and Biofilm Formation in Enterococci Isolated from Rhesus Macaques (*Macaca mulatta*): A Potential Threat for Wildlife in Bangladesh?

**DOI:** 10.3390/ani13142268

**Published:** 2023-07-11

**Authors:** Farhana Binte Ferdous, Md. Saiful Islam, Md. Ashek Ullah, Md. Liton Rana, Sadia Afrin Punom, Fahim Haque Neloy, Mohammad Nizam Uddin Chowdhury, Jayedul Hassan, Mahbubul Pratik Siddique, Sukumar Saha, Md. Tanvir Rahman

**Affiliations:** 1 Department of Microbiology and Hygiene, Faculty of Veterinary Science, Bangladesh Agricultural University, Mymensingh 2202, Bangladesh; farhanaferdous1501184@gmail.com (F.B.F.); dvm41257@bau.edu.bd (M.S.I.); ashek.21110216@bau.edu.bd (M.A.U.); liton.21110215@bau.edu.bd (M.L.R.); punom.vmh@bau.edu.bd (S.A.P.); neloymb002@gmail.com (F.H.N.); dr_jahid@bau.edu.bd (J.H.); mpsiddique@bau.edu.bd (M.P.S.); sukumar.saha@bau.edu.bd (S.S.); 2 Sheikh Kamal Wildlife Center, Bangladesh Forest Department, Gazipur 1706, Bangladesh; nizamvet05@gmail.com

**Keywords:** rhesus macaques, *Enterococcus faecalis*, *Enterococcus faecium*, antibiotic resistance, multidrug resistance, virulence factors, biofilm formation, Bangladesh

## Abstract

**Simple Summary:**

In this study, 67 rectal swab samples of rhesus macaques were screened to investigate the prevalence of antimicrobial resistance, virulence factors, and biofilm formation in enterococci found in rhesus macaques (*Macaca mulatta*) in Bangladesh. *Enterococcus faecalis* and *E. faecium* were detected in 65.7% and 22.4% of the samples; each of them was a biofilm former. The enterococci isolates showed phenotypic resistance to multiple antibiotics, including penicillin, rifampin, ampicillin, erythromycin, vancomycin, and linezolid. Moreover, multidrug resistance was exhibited in 88.63 % of *E. faecalis* and 100% of *E. faecium* isolates. The resistance *bla*_TEM_ gene was harbored in 61.4% and 60% of *E. faecalis* and *E. faecium* isolates, respectively. Virulence genes, such as *agg*, *fsrA*, *fsrB*, *fsrC*, *gelE*, *sprE*, *pil*, and *ace*, were harbored in enterococci isolates. As we know, this is the first report on determining antimicrobial resistance, virulence factors, and biofilm formation in enterococci from rhesus macaques in Bangladesh. The findings of this study highlight the potential threat of enterococci in rhesus macaques and their potential transmission to other wildlife species and humans in Bangladesh.

**Abstract:**

Enterococci are commensal bacteria that inhabit the digestive tracts of animals and humans. The transmission of antibiotic-resistant genes through human–animal contact poses a potential public health risk worldwide, as zoonoses from wildlife reservoirs can occur on every continent. The purpose of this study was to detect *Enterococcus* spp. in rhesus macaques (*Macaca mulatta*) and to investigate their resistance patterns, virulence profiles, and biofilm-forming ability. Conventional screening of rectal swabs (*n* = 67) from macaques was followed by polymerase chain reaction (PCR). The biofilm-forming enterococci were determined using the Congo red agar plate assay. Using the disk diffusion test (DDT), antibiogram profiles were determined, followed by resistance and virulence genes identification by PCR. PCR for bacterial species confirmation revealed that 65.7% (44/67) and 22.4% (15/67) of the samples tested positive for *E. faecalis* and *E. faecium*, respectively. All the isolated enterococci were biofilm formers. In the DDT, enterococcal isolates exhibited high to moderate resistance to penicillin, rifampin, ampicillin, erythromycin, vancomycin, and linezolid. In the PCR assays, the resistance gene *bla*_TEM_ was detected in 61.4% (27/44) of *E. faecalis* and 60% (9/15) of *E. faecium* isolates. Interestingly, 88.63 % (39/44) of *E. faecalis* and 100% (15/15) of *E. faecium* isolates were phenotypically multidrug-resistant. Virulence genes (*agg*, *fsrA*, *fsrB*, *fsrC*, *gelE*, *sprE*, *pil*, and *ace)* were more frequent in *E. faecalis* compared to *E. faecium*; however, isolates of both *Enterococcus* spp. were found negative for the *cyl* gene. As far as we know, the present study has detected, for the first time in Bangladesh, the presence of virulence genes in MDR biofilm-forming enterococci isolated from rhesus macaques. The findings of this study suggest employing epidemiological surveillance along with the one-health approach to monitor these pathogens in wild animals in Bangladesh, which will aid in preventing their potential transmission to humans.

## 1. Introduction

Wildlife has been an important source of human-transmissible infectious diseases throughout history. Zoonosis in a wildlife reservoir is a significant global public health concern [1]. Primates maintain the balance of structures and functions of ecosystems by pollinating plants, dispersing seeds, and acting as herbivores and predators [2]. Rhesus macaques (*Macaca mulatta*) are synanthropic and flourish in human-altered surroundings, which makes them one of the most wide-ranging and successful primates [3]. They prefer forested areas more for their maintenance. In the same way, different eco and safari parks provide breeding-friendly and natural environments that help them to adapt easily to these areas. Macaques are found in many urban, rural, and forested/protected areas in Bangladesh [4].

Given that forests are the natural habitats of primates, most human-primate interactions take place in these high-risk interfaces. In various parts of the world, primate species with omnivorous diets are adjusting to human activities. Moreover, the frequency of these interactions has risen due to factors, such as eco-tourism and the expansion of forest areas, potentially resulting in the exchange of bacteria through multiple routes, including the provision of food [5]. It is evident that monkeys, including rhesus macaques, can carry different bacterial agents, including *Enterococcus* spp., *Escherichia coli*, *Salmonella* spp., *Staphylococcus* spp., *Klebsiella* spp., *Campylobacter* spp., *Shigella* spp., and others [4,5,6,7].

Enterococci are commensal bacteria that inhabit the digestive tracts of a wide range of animals, from invertebrates to humans. Over 50 enterococcal species have been found, although *Enterococcus faecium* and *Enterococcus faecalis* are the most frequent in humans and animals [8]. Around 95% of all enterococcal infections are caused by these two species [9]. These bacteria are regarded as major nosocomial pathogens and markers of feces contamination. Approximately 80.36% and 8.93% of human diseases are caused by *E. faecalis* and *E. faecium*, respectively [10]. In humans, enterococci are able to cause a variety of human disorders, including urinary tract infections, endocarditis, meningitis, and sepsis [11]. 

Bacteria commonly employ biofilms as a strategy to survive challenging environmental conditions. Biofilms are formed when microbial cells aggregate and are enveloped by exopolymeric substances. Compared to individual planktonic cells, bacteria that form biofilms own several advantages, including greater resilience against environmental stress conditions, sanitizers, and antimicrobial agents [12]. Biofilms are clinically significant, as they account for more than 80% of microbial infections in the body [13]. Enterococci are recognized for their capacity to create biofilms, which consist of populations of cells firmly attached to diverse living and non-living surfaces [14]. Numerous virulence genes found in enterococci have been extensively examined due to their significant contributions to synthesize proteins involved in the development of biofilms. These genes include *agg* (aggregation substances); *fsrA*, *fsrB*, and *fsrC* (faecal streptococci regulators); *gelE* (gelatinase); *sprE* (serine protease); *ace* (collagen adhesion of *E. faecalis*); *pil* (endocarditis and biofilm-associated pili protein); *cyl* (cytolysin); and others [14,15,16,17].

Antimicrobial resistance (AMR) poses a significant and growing public health concern and presents a substantial obstacle to human and veterinary medicine. The increasing prevalence of AMR is becoming a greater concern in public health, as it diminishes the efficacy of antibiotics and makes the treatment of bacterial infections more challenging [18]. If left uncontained by the year 2050, AMR is projected to cause significant consequences, including hundreds of millions of human deaths, substantial economic setbacks, and a significant decline in livestock productivity [19]. The excessive use of antibiotics creates selective pressure, which serves as a significant driving force behind the emergence of resistance [20]. In Bangladesh, it is common for people to self-administer antimicrobial drugs without seeking guidance from licensed doctors or veterinarians to treat themselves or others, including animals [4]. The genes responsible for bacterial resistance, commonly found in the microbiota of humans or animals, are now being transmitted to natural environments and wild animals that have not been exposed to antimicrobial agents before [21]. Many bacteria that reside in both commensal organisms and the environment, including enterococci, exhibit a high prevalence of multidrug-resistant (MDR) genes [22]. Therefore, it is crucial to adopt a one-health approach to identify the various factors, including human, animal, and environmental aspects, that contribute to the rising levels of AMR [23]. The rapid emergence and dissemination of AMR in enterococci have become a significant public health hazard [24]. The treatment of enterococcal infections can become difficult due to the intrinsic resistance ability of these organisms against multiple antimicrobial categories, such as cephalosporins, aminoglycosides, macrolides, and sulfonamides [25].

The dynamics of AMR play a relevant role in the well-being of humans, animals, and the environment by contributing to the rise of zoonotic diseases and microorganisms. However, our understanding of AMR studies in wildlife is currently limited. Moreover, the majority of studies on enterococci in Bangladesh focused on cattle, poultry, fish, and humans [26,27,28,29,30,31,32,33], but the role of wild animals carrying enterococci is still being neglected. Previously, Islam et al. [34] detected enterococci from wild migratory birds. However, as far as we know, there is no study in Bangladesh determining antibiotic resistance, virulence, and biofilm-forming abilities in enterococci isolated from rhesus macaques. Therefore, this study was conducted to fill the aforementioned gaps.

## 2. Materials and Methods

### 2.1. Sample Information

**Figure 1:**
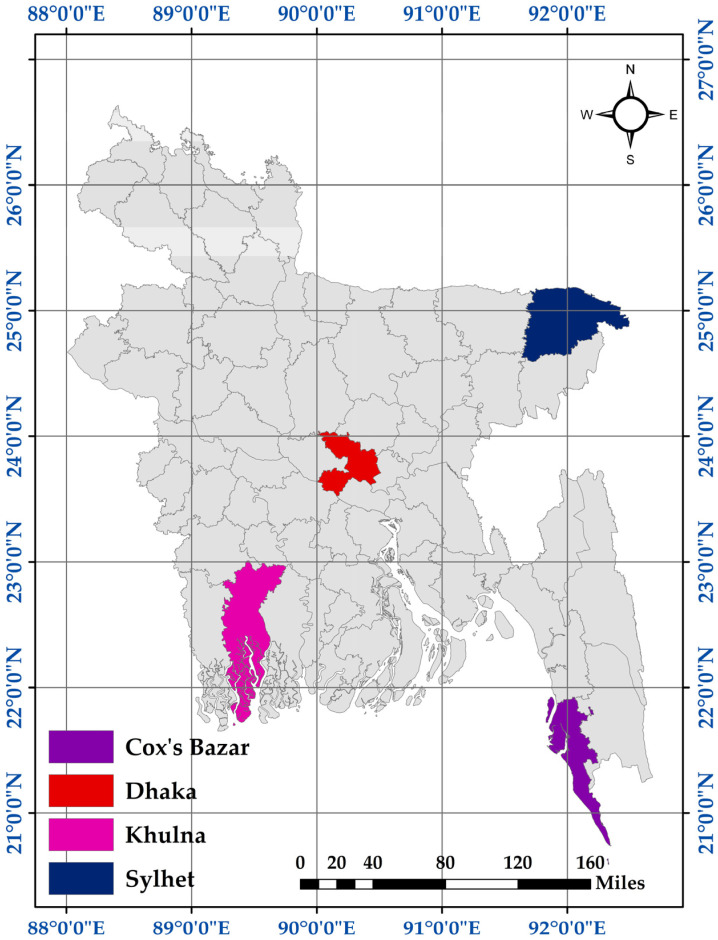
The map of the sampling sites during this study. The study area map was prepared using ArcMap.v.10.7 (ArcGIS Enterprise, ESRI, Redlands, CA, USA).

A total of 67 freshly collected rectal swab samples were obtained during the period of July 2022 to March 2023 from another ongoing project led by Dr. Nizam Uddin, Bangladesh Forest Department, Ministry of Environment, Forests & Climate Change, Dhaka, Bangladesh, on tuberculosis surveillance in wild animals. The samples were originally collected from five different districts of Bangladesh, including Safari Park, Cox’s Bazar (21.4339° N, 91.9870° E), Karamjal Wildlife Breeding Center, Khulna (22.4285° N, 89.5880° E), Sylhet Urban Area (24.9035° N, 91.8736° E), Tilagar Eco-park, Sylhet (24.9174° N, 91.9037° E), and Madaripur, Dhaka (23.1683° N, 90.1520° E) (Figure 1).

### 2.2. Isolation of Enterococci

After the arrival of the samples at the laboratory, they were incubated in a nutrient broth at 37 °C for 18 to 24 h. The nutrient broth, containing bacterial cultures, was then spread on Enterococcus agar base (EAB) media (HiMedia, Mumbai, Maharashtra, India) and incubated at 37 °C for 18–24 h. The enterococci, i.e., *E. faecalis* and *E. faecium*, were distinguished based on various phenotypic characteristics, including size, number, shape, and color of colonies on EAB media, growth capacity at 45 °C, Gram-staining properties, and characteristics in different biochemical tests, such as sugar fermentation, catalase, bile aesculin reaction, indole, and Voges–Proskauer tests [35].

### 2.3. Molecular Detection of Enterococci

*Enterococcus faecalis* and *E. faecium* were further confirmed by polymerase chain reaction (PCR) using species-specific *ddl_E. faecalis_* and *ddl_E. faecium_* primers, respectively (Table 1).

The PCR procedure involved extracting genomic DNA from isolated enterococci using the boiling method as described previously [38]. In summary, a 1 mL portion of the enriched culture was initially centrifuged at 5000 rpm for 5 min. The resulting supernatant was discarded, and a suspension was prepared by adding 200 μL of phosphate buffer solution. The next steps involved boiling and subsequent cooling of the suspension for 10 min, followed by centrifugation at 10,000 rpm for 10 min. The resulting supernatant, containing the genomic DNA, was collected and stored at −20 °C for further research.

All the PCR assays were carried out in a final volume of 20 µL reaction mixture containing 3 µL of nuclease-free water, 10 µL of the master mixture (Promega, Madison, WI, USA), 1 µL of forward primer, 1 µL of reverse primer, and 5 µL of DNA template. Once the amplification process was completed, the PCR products were observed by subjecting them to electrophoresis on a 1.5% agarose gel. Subsequently, the gel was stained with ethidium bromide and documented using an ultraviolet trans-illuminator (Biometra, Göttingen, Germany). To verify the expected size of the amplified PCR products, a 100 bp and 1 kb DNA ladder (Promega, Madison, WI, USA) were used as reference markers.

### 2.4. Biofilm Formation Capability of Enterococci

The biofilm-forming ability of enterococci was evaluated phenotypically using the Congo red (CR) test, as described previously [39]. In the CR assay, enterococci strains were cultured on Congo red agar (CRA) plates to determine their biofilm-forming abilities. In order to prepare CRA plates, 0.8 g of CR (HiMedia, Maharashtra, India) and 36 g of sucrose (HiMedia, Maharashtra, India) were mixed with 1000 mL of blood agar (HiMedia, Maharashtra, India). The mixture was then incubated at 37 °C overnight to ensure its sterility. Subsequently, enterococci cultures grown overnight were streaked onto CRA plates and incubated at 37 °C for 24 and 48 h. The observable characteristics of the examined isolates were then analyzed to assess their ability to form biofilms. Isolates displaying dry filamentous crusty black colonies, darkening but lacking the presence of dry crystalline structured colonies, almost black colonies, and red colonies were interpreted as strong, intermediate/moderate, weak, and non-biofilm formers, respectively [40,41].

### 2.5. Detection of Virulence Genes in Enterococci

The virulence-related genes commonly found in enterococci, e.g., *agg*, *fsrA*, *fsrB*, *fsrC*, *gelE*, *sprE*, *ace*, *pil*, and *cyl*, were detected by simplex PCR assay. Table 1 provides an overview of the primer sequences, PCR product sizes, and the corresponding references. The PCR amplification of virulence genes in enterococci was conducted using the same methodology employed for the detection of enterococci-specific genes.

### 2.6. Antimicrobial Susceptibility Test

Following the guidelines of CLSI [42], the antibiotic susceptibility patterns of enterococci to antimicrobial agents were determined using the disk diffusion method [43]. Thirteen commercially available antibiotics under ten antibiotic classes were selected in the current study, including penicillins (penicillin −10 μg and ampicillin −10 μg), glycopeptides (vancomycin −30 μg), macrolides (erythromycin −15 μg), tetracyclines (tetracycline -30 μg), fluoroquinolones (ciprofloxacin −5 μg, levofloxacin −5 μg, and norfloxacin −10 μg), nitrofurans (nitrofurantoin -300 μg), ansamycins (rifampin -5 μg), phosphonic acids (fosfomycin −200 μg), amphenicols (chloramphenicol −30 μg), and oxazolidinones (linezolid −30 μg). After being cultivated on EAB agar plates for a duration of 18–24 h, a suspension of 2–3 bacterial colonies was suspended in 0.85% sterile normal saline solution. This suspension was adjusted to a final concentration of 0.5 McFarland standard units for the antibiotic susceptibility test. After an additional 24 h incubation at 37 °C, the bacterial inoculum was spread onto Mueller–Hinton agar plates using sterile cotton swabs, and specific antibiotics were added to the plates as per selection. Isolates that exhibited resistance to a minimum of three antimicrobial categories were classified as MDR [44]. The calculation of the multiple antibiotic resistance (MAR) indices was performed using the following formula: MAR = u/v [45], where “u” represents the number of antibiotics to which an isolate displayed resistance, and “v” represents the total number of antibiotics employed in this study.

### 2.7. Statistical Analysis

Data obtained from this study were incorporated using Excel 365 (Microsoft/Office 365, Redmond, WA, USA); subsequently, analyses were performed in the Statistical Package for Social Science (SPSS.v.25, IBM, Chicago, IL, USA) and GraphPad Prism (Prism.v.8.4.2, San Diego, CA, USA).

A descriptive analysis was utilized to determine the prevalence of various variables. To estimate the prevalence, a binomial 95% confidence interval (CI) was calculated using a previous method [46] implemented in GraphPad Prism. The chi-square test for relatedness (Z-test for proportion) was performed to determine if there were any variances in the frequencies of enterococci isolates. Furthermore, a similar test was conducted to assess the differences in the occurrence of virulence and antibiotic resistance in relation to the various degrees of biofilm formation among enterococci isolates. Statistical significance was indicated by a *p*-value less than 0.05 (*p* < 0.05). A bivariate analysis using SPSS was also conducted to assess the potential correspondence among the virulence genes of enterococci isolates with a *p*-value of less than 0.05.

## 3. Results

### 3.1. Occurrence of Enterococci

Out of 67 samples, enterococci were found in 48 (71.6%; 95% CI: 59.9–81.0%) rectal swab samples of rhesus macaque. Among them, 44 (65.7%; 95% CI: 53.7–75.9%) were found to be positive for *E. faecalis*, and 15 (22.4%; 95% CI: 14.1–33.7%) were positive for *E. faecium* confirmed by PCR. Both *E. faecalis* and *E. faecium* were detected in 11 samples (16.4%, 95% CI: 9.4–27.1%). In addition, the prevalence of *E. faecalis* was significantly higher than that of *E. faecium* (*p* < 0.001).

### 3.2. Characteristics of Enterococcus faecalis

All the characteristic features of *E. faecalis* isolated in this study from rhesus macaque are documented in Table 2.

#### 3.2.1. Biofilm-Forming Ability of *E. faecalis*

The biofilm assay revealed all the *E. faecalis* as biofilm formers. The occurrence rate of intermediate biofilm-forming isolates (26/44, 59.1%, 95% CI: 44.4–72.3%) was significantly (*p* < 0.001) higher than strong (9/44, 20.5%, 95% CI: 11.2–34.5%) and weak (9/44, 20.5%, 95% CI: 11.2–34.5%) biofilm-forming *E. faecalis* isolates (Table 2).

#### 3.2.2. Occurrence of Virulence Genes in *E. faecalis*

In PCR, all the *E. faecalis* isolates harbored at least five investigated virulence genes. The highest number of *E. faecalis* isolates contained virulence genes *fsrC* and *sprE* (43/44, 97.7%, 95% CI: 88.2–99.9%), followed by *agg* and *pil* (42/44, 95.5%, 95% CI: 84.9–99.2%), *fsrB* and *sprE* (41/44, 93.2%, 95% CI: 81.77–97.65%), and *fsrA* and *ace* (35/44, 79.5%, 95% CI: 65.5–88.9%). No *E. faecalis* isolates harbored the virulence *cyl* gene (Table 2).

The bivariate analysis revealed a strong positive and significant correlation between virulence genes *fsrA* and *ace* (ρ = 0.871), *fsrB* and *sprE* (ρ = 0.482), *fsrA* and *pil* (ρ = 0.402), and *pil* and *ace* (ρ =0.402) (Supplementary Table S1).

Moreover, the occurrence of virulence genes, i.e., *agg*, *fsrA*, *pil*, and *ace*, was significantly associated with different degrees of biofilm formation in *E. faecalis* isolates. All the strong biofilm-forming isolates contained all the tested virulence genes except the *cyl* gene (Supplementary Table S2).

#### 3.2.3. Antibiogram Profiles of *E. faecalis*

In the disk diffusion method, all the 44 *E. faecalis* isolates (95% CI: 91.97–100%) were resistant to penicillin, ampicillin, and rifampin, followed by linezolid and erythromycin (59.1%, 26/44, 95% CI: 44.4–72.3%), vancomycin (52.3%, 23/44, 95% CI: 37.9–66.2%), tetracycline (20.5%, 9/44, 95% CI: 11.2–34.5%), and chloramphenicol (6.8%, 3/44, 95% CI: 2.4–18.2%) (Table 2). There was no resistance to ciprofloxacin, levofloxacin, norfloxacin, nitrofurantoin, and fosfomycin.

Interestingly, 88.6% (39/44, 95% CI: 86.0–95.1%) of the *E. faecalis* isolates were phenotypically MDR in nature, where the MAR indices varied from 0.2 to 0.6. Sixteen antibiotic resistance patterns were observed; among them, 15 showed multidrug resistance. One isolate exhibited resistance to eight antibiotics under seven antibiotics classes (Table 3). Moreover, in PCR, 27 (61.4%, 95% CI: 46.6–74.3%) *E. faecalis* isolates contained the resistance *bla*_TEM_ gene (Table 2).

A statistically significant association was observed between the degrees of biofilm formation and the resistance profiles of *E. faecalis* isolates against linezolid, vancomycin, erythromycin, and the resistant *bla*_TEM_ gene (Supplementary Table S3).

### 3.3. Characteristics of Enterococcus faecium

All the characteristic features of *E. faecium* isolated in this study from rhesus macaque are documented in Table 4.

#### 3.3.1. Biofilm-Forming Ability of *E. Faecium*

In the CR assay method, the significantly (*p* < 0.05) higher *E. faecium* isolates were intermediate biofilm formers (9/15, 60%, 95% CI: 35.8–80.2%), followed by strong (3/15, 20%, 95% CI: 7.0–45.2%) and weak (3/15, 20%, 95% CI: 7.0–45.2%) biofilm formers (Table 4).

#### 3.3.2. Occurrence of Virulence Genes in *E. faecium*

In PCR, five virulence genes, i.e., *fsrA*, *fsrB*, *fsrC*, *gelE*, and *sprE*, were harbored in 40% (6/15, 95% CI: 19.8–64.3%) of the *E. faecium* isolates. In addition, virulence genes *pil* and *ace* were found in 26.7% (4/15, 95% CI: 10.9–51.9%) and *agg* in 13.3% (2/15, 95% CI: 2.4–37.9%) of the isolates. The virulence gene *cyl* was not detected in any *E. faecium* isolates (Table 4).

Moreover, the bivariate analysis revealed a strong positive and significant correlation among virulence genes of *E. faecium*, such as *ace* and *agg* (ρ = 0.656), *ace* and *fsrA* (ρ = 0.739), *ace* and *fsrB* (ρ = 0.739), *ace* and *fsrC* (ρ = 0.739), *ace* and *gelE* (ρ = 0.739), *ace* and *sprE* (ρ = 0.739), *fsrA* and *fsrB* (ρ = 0.722), *fsrA* and *fsrC* (ρ = 0.722), *fsrB* and *fsrC* (ρ = 0.722), *fsrA* and *gelE* (ρ = 0.722), *fsrB* and *gelE* (ρ = 0.722), *fsrC* and *gelE* (ρ = 0.722), and *cyl* and *ace* (ρ = 0.650) (Supplementary Table S4).

A statistically significant relationship was exhibited between the biofilm-forming ability and the occurrence of virulence genes, i.e., *agg*, *fsrA*, *fsrB*, *fsrC*, *gelE*, *sprE*, and *ace*, in *E. faecium* isolates. The detection rate of all virulence genes was higher in strong biofilm-forming *E. faecium* isolates (Supplementary Table S5).

#### 3.3.3. Antibiogram Profiles of *E. faecium*

In the antimicrobial susceptibility test, 100% (15/15, 95% CI: 79.6–100%) of the *E. faecium* isolates showed resistance to penicillin, ampicillin, rifampin, and erythromycin, 60% (9/15, 95% CI: 35.4–80.2%) to linezolid, and 46.7% (7/15, 95% CI: 24.8–69.9%) to vancomycin (Table 4). All the isolates were sensitive to ciprofloxacin, norfloxacin, levofloxacin, chloramphenicol, tetracycline, fosfomycin, and nitrofurantoin.

Surprisingly, phenotypic multidrug resistance characteristics were observed in all the *E. faecium* isolates, and the MAR indices ranged between 0.3 and 0.5. Three resistance patterns were determined, where seven isolates were resistant to six antibiotics under five antibiotic classes (Table 3). Moreover, in the PCR assays, the resistant gene *bla*_TEM_ was detected in 60% (9/15, 95% CI: 35.8–80.2%) of the *E. faecium* isolates (Table 4).

Moreover, the degrees of biofilm formation and resistance patterns of *E. faecium* isolates against linezolid, vancomycin, and the resistant *bla*_TEM_ gene showed a statistically significant association (Supplementary Table S6).

## 4. Discussion

The acquisition of resistant bacterial strains from wild animals can disclose key aspects of microbial interactions and environmental perturbations in wildlife [47]. In Bangladesh, rhesus macaques are directly or indirectly associated with humans, other animals, and environments, posing an opportunity to become potential drivers of resistant pathogen transmission. This study was undertaken to evaluate the level of antimicrobial resistance, virulence, and biofilm formation in enterococci isolated from rhesus macaques in Bangladesh having potential significant issues for public health.

In this study, 71.6% of the samples contained enterococci, of which the detection rate of *E. faecalis* was more predominant than *E. faecium*. Previously, Grassotti et al. [5] detected *E. faecalis* and *E. faecium* from fecal and rectal swabs of macaques in Brazil in similar patterns. Moreover, several previous studies revealed that macaques could be an important source of enterococci [48,49,50,51]. The presence of enterococci in free-ranging rhesus macaques suggests that these macaques might be infected through contact with infected humans and animals or contaminated environments. Moreover, during the observations at the collection sites, both direct and indirect interaction between macaques, humans, animals, and the surrounding environment were evidenced during the presence study. This suggests a possible pathway for the transmission of enterococci. Previously, Rahman et al. [4] detected bacterial pathogens from rhesus macaques in Bangladesh; however, they did not detect enterococci. Enterococci found in rhesus macaques, especially free-ranging non-human primates have the potential to be transferred to humans and environments as these macaques are directly or indirectly associated with humans and environments for their foods. The intrusion of humans into the natural habitat of macaques could potentially be responsible for an elevated level of interaction between people and macaques, which suggests the transmission of enterococci from macaques to humans and vice versa.

Forming biofilms is a highly significant attribute of microorganisms that receives substantial focus in clinical microbiology [52]. The presence of a biofilm, or a slimy layer, on surfaces is linked to the production of extracellular polysaccharides, which plays a crucial role in the attachment of bacteria [53]. Despite not being the most sensitive method for assessing biofilm development, the researchers chose to utilize the CRA test due to its satisfactory levels of sensitivity and specificity [54]. In this study, all the enterococci were biofilm formers; among them, 20.5% of *E. faecalis* and 20% of *E. faecium* isolates were strong biofilm formers. The presence of biofilm-forming enterococci in rhesus macaques has a negative impact on human, animal, and environmental health since the biofilm-formation facilitates the development of antimicrobial resistance and virulence in bacterial pathogens [55]. Enterococci possess an exceptional capacity to develop biofilms, a unique approach for causing disease that enables their survival in challenging environments and the long-term presence at the site of infection [56]. As far as we know, there was no published data on the detection of biofilm-forming enterococci from rhesus macaques in Bangladesh.

The biofilm formation process is intricate and influenced by multiple factors, but it can be partially attributed to particular virulence factors, including those related to the ability of enterococci to adhere and colonize the host [57]. The remarkable biofilm formation capability observed in this research could be attributed to various examined genes associated with enterococci biofilm formation, as identified in numerous studies among the isolates [14,15,29]. Previously, Soares et al. [58] and Anderson et al. [59] showed that the ability of enterococci isolates to form biofilm is significantly associated with the presence of virulence genes. In our study, most of the enterococci isolated from rhesus macaques contained virulence genes, of which all the *E. faecalis* isolates contained virulence genes. By contrast, only two out of 15 *E. faecium* isolates did not harbor any tested virulence genes. The presence of a high prevalence of virulence genes in enterococci isolated from rhesus macaques suggests that rhesus macaques could be important sources for the transmission of these virulent enterococci to humans, animals, and the environment that need further investigation.

In addition, a considerable increase in virulence genes was observed in enterococci isolates that exhibited strong or intermediate biofilm formation. This suggests that, as the extent of biofilm creation in enterococci isolates rises, their capacity to initiate infections also grows. However, additional research is required to establish the precise correlation between the biofilm-forming capacity of enterococci isolates and their virulence genes since targeting virulence factors could serve as a strategy for developing new medications aimed at preventing bacterial biofilms [60]. Moreover, additional virulence factors, such as *asa1*, *efaA*, *esp*, and *ebp*, might not be examined in this study but could contribute to the strong biofilm-forming capability of the enterococci isolates.

Antimicrobial treatment is infrequent in wild animals, especially in free-ranging rhesus macaques; nonetheless, wildlife has been implicated as a potential reservoir of resistant bacteria and genes associated with resistance [61]. In the current study, enterococci isolated from free-ranging rhesus macaques showed high resistance to penicillin, ampicillin, rifampin, linezolid, erythromycin, and vancomycin. In addition, more than 60% of enterococci isolates harbored the beta-lactamase *bla*_TEM_ gene. Previously, Grassotti et al. [5] recorded that the enterococci isolated from macaques exhibited resistance to rifampicin, tetracycline, erythromycin, nitrofurantoin, chloramphenicol, and ampicillin; however, they did not find any vancomycin-resistant enterococci isolates. The presence of vancomycin- and linezolid-resistant enterococci isolates in rhesus macaques poses a serious threat to health communities since vancomycin and linezolid are antibiotics of last resort utilized to treat severe infections caused by MDR Gram-positive bacteria [62,63]. However, further studies using MIC determination and molecular approaches should be conducted before coming to such an important conclusion. Moreover, the presence of antibiotic-resistant enterococci strains in wild rhesus macaques raises concerns as these animals have not been subjected to therapeutic antibiotic treatment in the past. They can acquire resistant bacterial strains by means of contamination of the environment, especially feces-polluted water. Wastewater and manure from antibiotic-treated humans and animals release intestinal bacteria into the environment, and sewage and runoff from manure-fertilized fields enter rivers, allowing for the long-distance transport of fecal bacteria [64]. Moreover, wild rhesus macaques can act as sentinels for the appearance and spread of bacteria that are resistant to antibiotics [65]. Therefore, the presence of antibiotic-resistant enterococci in these animals highlights the influence of human activities on the development of antimicrobial resistance in environmental sources. However, it is crucial to acknowledge that wild rhesus macaques residing in freely accessible areas that are in close proximity to both human and animal environments can serve as a potential source of antibiotic-resistant enterococci isolates in these animals.

Despite antibiotic treatment and the host’s immune and inflammatory responses, biofilms persist within the host organism due to the reduced metabolic activity of biofilm cells, the occurrence of the quorum-sensing phenomenon, and distinct resistance mechanisms [66]. In this study, strong biofilm-forming enterococci isolates had higher resistance against most of the tested antibiotics, which suggests that the biofilm-forming abilities of enterococci isolates are related to the occurrence of antimicrobial resistance. According to Uruén et al. [67], bacteria having biofilm-forming capabilities showed increased resistance compared to bacteria without biofilm-forming abilities. The extraordinary resistance of biofilm-forming enterococci to antibiotics can be attributed to several factors, including the reduced metabolic and growth rates of biofilm formers, which inherently make them less susceptible to antibiotics; the composition of the biofilm extracellular polymeric substances matrix, which aids in limiting the penetration of antibiotics into biofilm regions; and the unique physiological traits of biofilm cells that facilitate the expression of multi-drug efflux pumps and stress-response regulons, enabling the development of antibiotic resistance [68,69].

In the present study, 88.6% of *E. faecalis* and 100% of *E. faecium* isolates showed multidrug resistance capabilities. The presence of MDR enterococci isolates observed in rhesus macaques can be attributed to the exchange and spread of resistant microorganisms among macaques, humans, and livestock through interactions among them. A previous study [5] demonstrated a noticeably decreased proportion of MDR strains (14.52%) linked to enterococci, in contrast to the findings of the present study. These differences can be attributed to various factors. These factors include the diverse range of enterococci observed in specific animal species, which may be influenced by factors, such as diet or the ecological niche they inhabit. Additionally, variations in the methods used to collect and isolate resistant strains (such as whether samples taken individually or in groups or the type of selective medium used) could contribute to the disparities. Furthermore, the extent to which each species has adapted to human environments and the level of urbanization in the region from which the animals originate may also play a role [70]. Collectively, all the enterococci isolates identified in this study exhibited a MAR index exceeding 0.2, indicating indiscriminate antibiotic usage at the contamination source. As suggested by Krumperman [45], isolates with a MAR index exceeding 0.2 are likely derived from a high-risk contamination source characterized by frequent antibiotic application. High numbers of MDR enterococci isolates with high MAR indices detected in rhesus macaques suggest the implementation of appropriate antimicrobial resistance surveillance in zoos and free-ranging areas where rhesus macaques stay.

The major limitations of this study were the small sample size and the use of non-randomized sampling. Access to wild animals for sampling is always tough and challenging; hence, these limitations could not be overcome.

## 5. Conclusions

Diseases caused by MDR zoonotic pathogens are major public health concerns. As far as we know, this study evaluated antibiotic resistance, biofilm-forming capabilities, and virulence determinants in enterococci isolated from rhesus macaques for the first time in Bangladesh. The findings of the present study suggest that rhesus macaques carry biofilm-forming MDR enterococci. Moreover, it suggests that these wild animals can act as environmental sentinels and vehicles of infections to other species, including humans. Therefore, this study highlights the importance of continuously monitoring the spread of antimicrobial-resistant, virulent, and biofilm-forming strains in these hosts to monitor environmental health, to provide useful tools for epidemiological assessments, and to timely foresee any emerging risks for humans. Further studies with the one-health approach are warranted to explore the mechanisms behind these observed traits and develop strategies for effective prevention and intervention to reduce the spread of AMR.

## Figures and Tables

**Table 1:** List of primers used in the current study.

Factors	Target Genes	Primer Sequences (5′–3′)	Annealing Tm (°C)	Size (bp)	Reference
*Enterococcus faecalis*	*ddl_E. faecalis_*	F: ATCAAGTACAGTTAGTCTTTA R: AACGATTCAAAGCTAACT	48	942	[36]
*Enterococcus faecium*	*ddl_E. faecium_*	F: GCAAGGCTTCTTAGAGA R: CATCGTGTAAGCTAACTTC	50	550	[36]
Virulence	*agg*	F: TCTTGGACACGACCCATGAT R: AGAAAGAACATCACCACGAGC	58	413	[14]
	*fsrA*	F: CGTTCCGTCTCTCATAGTTA R: GCAGGATTTGAGGTTGCTAA	53	474	[14]
	*fsrB*	F: TAATCTAGGCTTAGTTCCCAC R: CTAAATGGCTCTGTCGTCTAG	55	428	[14]
	*fsrC*	F: GTGTTTTTGATTTCGCCAGAGA R: TATAACAATCCCCAACCGTG	54	716	[14]
	*gelE*	F: GGTGAAGAAGTTACTCTGAC R: GGTATTGAGTTATGAGGGGC	52	704	[14]
	*sprE*	F: CTGAGGACAGAAGACAAGAAG R: GGTTTTTCTCACCTGGATAG	53	432	[14]
	*ace*	F: GAATGACCGAGAACGATGGC R: CTTGATGTTGGCCTGCTTCC	58	615	[14]
	*pil*	F: GAAGAAACCAAAGCACCTAC R: CTACCTAAGAAAAGAAACGCG	53	620	[14]
	*cyl*	F: TGGCGGTATTTTTACTGGAG R: TGAATCGCTCCATTTCTTC	52	186	[15]
Antibiotic resistance	*bla*_TEM_	F: CATTTCCGTGTCGCCCTTAT R: TCCATAGTTGCCTGACTCCC	56	793	[37]

**Table 2:** Biofilm-forming, virulence, and antibiotic resistance features of *E. faecalis* isolated from rhesus macaques in Bangladesh.

Sample ID	Biofilm Properties	Virulence Gene Pattern	Antibiogram Profiles	
			Phenotype	Genotype
RM-1	Intermediate	*agg*, *pil*, *fsrA*, *fsrB*, *fsrC*, *ace*, *sprE*, *gelE*	P, AMP, RD	*bla*_TEM_
RM-2	Intermediate	*agg*, *pil*, *fsrA*, *fsrB*, *fsrC*, *ace*, *sprE*, *gelE*	P, AMP, RD, E, LZD, TE, C	
RM-3	Weak	*agg*, *fsrB*, *fsrC*, *sprE*, *gelE*	P, AMP, RD	
RM-4	Intermediate	*agg*, *pil*, *fsrA*, *fsrB*, *fsrC*, *ace*, *sprE*, *gelE*	P, AMP, RD, E, VA, TE	
RM-5	Strong	*agg*, *pil*, *fsrA*, *fsrB*, *fsrC*, *ace*, *sprE*, *gelE*	P, AMP, RD, E, LZD, VA	*bla*_TEM_
RM-8	Intermediate	*agg*, *pil*, *fsrA*, *fsrB*, *fsrC*, *ace*, *sprE*, *gelE*	P, AMP, RD, E, LZD, VA	*bla*_TEM_
RM-10	Intermediate	*agg*, *pil*, *fsrA*, *fsrB*, *fsrC*, *ace*, *sprE*, *gelE*	P, AMP, RD, E, VA	
RM-11	Strong	*agg*, *pil*, *fsrA*, *fsrB*, *fsrC*, *ace*, *sprE*, *gelE*	P, AMP, RD, E, LZD, VA	*bla*_TEM_
RM-13	Strong	*agg*, *pil*, *fsrA*, *fsrB*, *fsrC*, *ace*, *sprE*, *gelE*	P, AMP, RD, E, LZD, VA	*bla*_TEM_
RM-15	Weak	*pil*, *fsrB*, *fsrC*, *sprE*, *gelE*	P, AMP, RD	*bla*_TEM_
RM-16	Strong	*agg*, *pil*, *fsrA*, *fsrB*, *fsrC*, *ace*, *sprE*, *gelE*	P, AMP, RD, E, LZD, VA	*bla*_TEM_
RM-17	Strong	*agg*, *pil*, *fsrA*, *fsrB*, *fsrC*, *ace*, *sprE*, *gelE*	P, AMP, RD, E, LZD, VA, C	*bla*_TEM_
RM-18	Intermediate	*agg*, *pil*, *fsrA*, *fsrB*, *fsrC*, *ace*, *sprE*, *gelE*	P, AMP, RD, E, LZD, VA	*bla*_TEM_
RM-19	Weak	*agg*, *pil*, *fsrB*, *fsrC*, *sprE*, *gelE*	P, AMP, RD	*bla*_TEM_
RM-20	Weak	*pil*, *fsrB*, *fsrC*, *sprE*, *gelE*	P, AMP, RD	
RM-21	Intermediate	*agg*, *pil*, *fsrA*, *fsrB*, *fsrC*, *ace*, *sprE*, *gelE*	P, AMP, RD, TE	*bla*_TEM_
RM-22	Intermediate	*agg*, *pil*, *fsrA*, *fsrB*, *fsrC*, *ace*, *sprE*, *gelE*	P, AMP, RD, E, LZD	*bla*_TEM_
RM-25	Intermediate	*agg*, *pil*, *fsrA*, *fsrB*, *fsrC*, *ace*, *sprE*	P, AMP, RD, LZD, VA	
RM-27	Intermediate	*agg*, *pil*, *fsrA*, *fsrC*, *ace*, *sprE*, *gelE*	P, AMP, RD, E, VA	*bla*_TEM_
RM-28	Weak	*agg*, *fsrB*, *fsrC*, *sprE*, *gelE*	P, AMP, RD, E	*bla*_TEM_
RM-29	Intermediate	*agg*, *pil*, *fsrA*, *fsrB*, *fsrC*, *ace*, *sprE*, *gelE*	P, AMP, RD, VA	
RM-31	Intermediate	*agg*, *pil*, *fsrA*, *fsrB*, *fsrC*, *ace*, *sprE*, *gelE*	P, AMP, RD, E, LZD, VA	*bla*_TEM_
RM-32	Intermediate	*agg*, *pil*, *fsrA*, *fsrB*, *fsrC*, *ace*, *sprE*, *gelE*	P, AMP, RD, LZD, VA	
RM-34	Strong	*agg*, *pil*, *fsrA*, *fsrB*, *fsrC*, *ace*, *sprE*, *gelE*	P, AMP, RD, E, LZD, VA	*bla*_TEM_
RM-36	Intermediate	*agg*, *pil*, *fsrA*, *fsrB*, *fsrC*, *ace*, *sprE*	P, AMP, RD, E, LZD	
RM-37	Weak	*agg*, *pil*, *fsrB*, *fsrC*, *sprE*, *gelE*	P, AMP, RD, VA	*bla*_TEM_
RM-39	Strong	*agg*, *pil*, *fsrA*, *fsrB*, *fsrC*, *ace*, *sprE*, *gelE*	P, AMP, RD, E, LZD, VA	*bla*_TEM_
RM-40	Intermediate	*agg*, *pil*, *fsrA*, *fsrB*, *fsrC*, *ace*, *sprE*, *gelE*	P, AMP, RD, E, LZD	
RM-41	Weak	*agg*, *pil*, *fsrB*, *fsrC*, *sprE*, *gelE*	P, AMP, RD, E, TE	*bla*_TEM_
RM-42	Weak	*agg*, *pil*, *fsrB*, *fsrC*, *sprE*, *gelE*	P, AMP, RD, LZD	
RM-43	Strong	*agg*, *pil*, *fsrA*, *fsrB*, *fsrC*, *ace*, *sprE*, *gelE*	P, AMP, RD, E, LZD, VA, TE	*bla*_TEM_
RM-44	Intermediate	*agg*, *pil*, *fsrA*, *fsrB*, *fsrC*, *ace*, *sprE*	P, AMP, RD, E, LZD	
RM-45	Intermediate	*agg*, *pil*, *fsrA*, *fsrB*, *ace*, *sprE*, *gelE*	P, AMP, RD, VA, TE	
RM-46	Intermediate	*agg*, *pil*, *fsrA*, *fsrC*, *ace*, *sprE*, *gelE*	P, AMP, RD, LZD, VA	*bla*_TEM_
RM-48	Intermediate	*agg*, *pil*, *fsrA*, *fsrB*, *fsrC*, *ace*, *sprE*, *gelE*	P, AMP, RD, E, TE	*bla*_TEM_
RM-50	Intermediate	*agg*, *pil*, *fsrA*, *fsrB*, *fsrC*, *ace*, *sprE*, *gelE*	P, AMP, RD, LZD	
RM-51	Intermediate	*agg*, *pil*, *fsrA*, *fsrB*, *fsrC*, *ace*, *sprE*, *gelE*	P, AMP, RD, E, TE	*bla*_TEM_
RM-55	Intermediate	*agg*, *pil*, *fsrA*, *fsrB*, *fsrC*, *ace*, *sprE*, *gelE*	P, AMP, RD, VA	
RM-56	Strong	*agg*, *pil*, *fsrA*, *fsrB*, *fsrC*, *ace*, *sprE*, *gelE*	P, AMP, RD, E, LZD, VA, TE, C	*bla*_TEM_
RM-57	Intermediate	*agg*, *pil*, *fsrA*, *fsrC*, *ace*, *sprE*, *gelE*	P, AMP, RD, LZD	
RM-61	Intermediate	*agg*, *pil*, *fsrA*, *fsrB*, *fsrC*, *ace*, *sprE*, *gelE*	P, AMP, RD, E, LZD	*bla*_TEM_
RM-62	Weak	*agg*, *pil*, *fsrB*, *fsrC*, *gelE*	P, AMP, RD, E	*bla*_TEM_
RM-63	Intermediate	*agg*, *pil*, *fsrA*, *fsrB*, *fsrC*, *ace*, *sprE*, *gelE*	P, AMP, RD, LZD	
RM-66	Intermediate	*agg*, *pil*, *fsrA*, *fsrB*, *fsrC*, *ace*, *sprE*, *gelE*	P, AMP, RD, LZD, VA	*bla*_TEM_

Here, RM = rhesus macaque, P = penicillin, AMP = ampicillin, RD = rifampin, E = erythromycin, LZD = linezolid, VA = vancomycin, C = chloramphenicol, TE = tetracycline.

**Table 3:** Multidrug resistance and multiple antibiotic resistance profiles of enterococci isolates detected from rectal swab samples of rhesus macaques.

No. of Pattern	Antibiotic Resistance Patterns	No. of Antibiotics (Classes)	No. of Isolates	Overall MDR Isolates (%)	MAR Index
*Enterococcus faecalis*					
1	P, AMP, RD, E, LZD, VA, TE, C	8 (7)	1	39/44 (88.6)	0.6
2	P, AMP, RD, E, LZD, TE, C	7 (6)	1		0.5
3	P, AMP, RD, E, LZD, VA, C	7 (6)	1		0.5
4	P, AMP, RD, E, LZD, VA, TE	7 (6)	1		0.5
5	P, AMP, RD, E, VA, TE	6 (5)	1		0.5
6	P, AMP, RD, E, LZD, VA	6 (5)	9		0.5
7	P, AMP, RD, E, LZD	5 (4)	5		0.4
8	P, AMP, RD, LZD, VA	5 (4)	4		0.4
9	P, AMP, RD, E, VA	5 (4)	2		0.4
10	P, AMP, RD, VA, TE	5 (4)	1		0.4
11	P, AMP, RD, E, TE	5 (4)	3		0.4
12	P, AMP, RD, LZD	4 (3)	4		0.3
13	P, AMP, RD, TE	4 (3)	1		0.3
14	P, AMP, RD, E	4 (3)	2		0.3
15	P, AMP, RD, VA	4 (3)	3		0.3
16 *	P, AMP, RD	3 (2)	5 *		0.2
***Enterococcus faecium***					
1	P, AMP, RD, E, LZD, VA	6 (5)	7	15/15 (100)	0.5
2	P, AMP, RD, E, LZD	5 (4)	2		0.4
3	P, AMP, RD, E	4 (3)	6		0.3

Here, MDR = multidrug-resistant, MAR = multiple antibiotic resistance, P = penicillin, AMP = ampicillin, RD = rifampin, E = erythromycin, LZD = linezolid, VA = vancomycin, C = chloramphenicol, TE = tetracycline, * = non-multidrug-resistant.

**Table 4:** Biofilm-forming, virulence, and antibiotic resistance features of *E. faecium* isolated from rhesus macaque in Bangladesh.

Sample ID	Biofilm Properties	Virulence Gene Pattern	Antibiogram Profile	
			Phenotype	Genotype
RM-4	Intermediate	*pil*	P, AMP, RD, E	
RM-10	Weak	-	P, AMP, RD, E	
RM-16	Strong	*fsrA*, *fsrB*, *fsrC*, *ace*, *sprE*, *gelE*	P, AMP, RD, E, LZD, VA	*bla*_TEM_
RM-17	Intermediate	*fsrB*	P, AMP, RD, E	*bla*_TEM_
RM-23	Intermediate	*pil*, *sprE*	P, AMP, RD, E, LZD, VA	
RM-27	Intermediate	*pil*, *sprE*	P, AMP, RD, E, LZD, VA	*bla*_TEM_
RM-29	Intermediate	*gelE*	P, AMP, RD, E, LZD	*bla*_TEM_
RM-36	Intermediate	*fsrC*	P, AMP, RD, E, LZD, VA	*bla*_TEM_
RM-37	Intermediate	*fsrA*	P, AMP, RD, E	
RM-39	Strong	*agg*, *pil*, *fsrA*, *fsrB*, *fsrC*, *ace*, *sprE*, *gelE*	P, AMP, RD, E, LZD, VA	*bla*_TEM_
RM-42	Weak	-	P, AMP, RD, E	
RM-43	Intermediate	*fsrA*, *fsrB*, *fsrC*, *gelE*	P, AMP, RD, E, LZD, VA	*bla*_TEM_
RM-52	Intermediate	*fsrA*, *fsrB*, *fsrC*, *ace*, *sprE*, *gelE*	P, AMP, RD, E, LZD	*bla*_TEM_
RM-59	Strong	*agg*, *fsrA*, *fsrB*, *fsrC*, *ace*, *sprE*, *gelE*	P, AMP, RD, E, LZD, VA	*bla*_TEM_
RM-67	weak	-	P, AMP, RD, E	

Here, RM = rhesus macaque, P = penicillin, AMP = ampicillin, RD = rifampin, E = erythromycin, LZD = linezolid, VA = vancomycin.

## Data Availability

All the data are available in the manuscript and Supplementary Material File.

## Supplementary Materials

The following supporting information can be downloaded at: https://www.mdpi.com/article/10.3390/ani13142268/s1, Table S1: Pearson correlation coefficient to assess the pairs of any of the two virulence genes detected in *E. faecalis* isolated from rectal swab samples of rhesus macaques; Table S2: Association in the detection of virulence genes and determination of biofilm formation in *E. faecalis* (*n* = 44) isolated from rectal swab samples of rhesus macaques; Table S3: Association of antibiotic resistance patterns and biofilm formation in *E. faecalis* strains detected in rectal swab samples of rhesus macaques; Table S4: Pearson correlation coefficient to assess the pairs of any of two virulence genes detected in *E. faecium* isolated from rectal swab samples of rhesus macaques; Table S5: Association in the detection of virulence genes and determination of biofilm formation in *E. faecium* (*n* = 15) isolated from rectal swab samples of rhesus macaques; Table S6: Association of antibiotic resistance patterns and biofilm formation in *E. faecium* strains detected in rectal swab samples of rhesus macaques.
