# Supplementary material for: Antimicrobial Resistance Profiles, Virulence Determinants, and Biofilm Formation in Enterococci Isolated from Rhesus Macaques (Macaca mulatta): A Potential Threat for Wildlife in Bangladesh?
Source: Animals (Basel). 2023 Jul 11;13(14):2268. doi: 10.3390/ani13142268 (PMC10376288; doi:10.3390/ani13142268)
Supplement: Supplementary file 1 [file animals-13-02268-s001.zip › animals-2489262-supplementary.pdf]

*Supplementary Materials*

# Antimicrobial Resistance Profiles, Virulence Determinants, and Biofilm Formation in Enterococci Isolated from Rhesus Macaques (*Macaca mulatta*): A Potential Threat for Wildlife in Bangladesh?

**Farhana Binte Ferdous [1], Md. Saiful Islam [1], Md. Ashek Ullah [1], Md. Liton Rana [1], Sadia Afrin Punom [1], Fahim Haque Neloy [1], Mohammad Nizam Uddin Chowdhury [2], Jayedul Hassan [1], Mahbubul Pratik Siddique [1], Sukumar Saha [1] and Md. Tanvir Rahman [1,*]**

[1] Department of Microbiology and Hygiene, Faculty of Veterinary Science, Bangladesh Agricultural University, Mymensingh 2202, Bangladesh; farhanaferdous1501184@gmail.com (F.B.F.); dvm41257@bau.edu.bd (M.S.I.); ashek.21110216@bau.edu.bd (M.A.U.); liton.21110215@bau.edu.bd (M.L.R.); punom.vmh@bau.edu.bd (S.A.P.); neloymb002@gmail.com (F.H.N.); dr_jahid@bau.edu.bd (J.H.); mpsiddique@bau.edu.bd (M.P.S.); sukumar.saha@bau.edu.bd (S.S.)

[2] Sheikh Kamal Wildlife Center, Bangladesh Forest Department, Gazipur 1706, Bangladesh; nizamvet05@gmail.com

* Correspondence: tanvirahman@bau.edu.bd

## Supplementary Materials

**Supplementary Table S1.** Pearson correlation coefficient to assess the pairs of any of two virulence genes detected in *E. faecalis* isolated from rectal swab of samples of rhesus macaques.

| | | *agg* | *fsrA* | *fsrB* | *fsrC* | *pil* | *gelE* | *sprE* | *ace* | *cyl* |
|---|---|---|---|---|---|---|---|---|---|---|
| *agg* | Pearson Correlation | 1 | | | | | | | | |
| | Sig. (2-tailed) | | | | | | | | | |
| *fsrA* | Pearson Correlation | 0.142 | 1 | | | | | | | |
| | Sig. (2-tailed) | 0.358 | | | | | | | | |
| *fsrB* | Pearson Correlation | -0.069 | 0.017 | 1 | | | | | | |
| | Sig. (2-tailed) | 0.656 | 0.912 | | | | | | | |
| *fsrC* | Pearson Correlation | -0.048 | -0.118 | -0.069 | 1 | | | | | |
| | Sig. (2-tailed) | 0.759 | 0.444 | 0.656 | | | | | | |
| *pil* | Pearson Correlation | -0.048 | 0.402** | -0.069 | -0.048 | 1 | | | | |
| | Sig. (2-tailed) | 0.759 | 0.007 | 0.656 | 0.759 | | | | | |
| *gelE* | Pearson Correlation | -0.059 | 0.068 | -0.086 | -0.059 | -0.059 | 1 | | | |
| | Sig. (2-tailed) | 0.703 | 0.659 | 0.581 | 0.703 | 0.703 | | | | |
| *sprE* | Pearson Correlation | -0.033 | 0.281 | 0.482** | -0.033 | -0.033 | -0.041 | 1 | | |
| | Sig. (2-tailed) | 0.83 | 0.064 | 0.001 | 0.83 | 0.83 | 0.79 | | | |
| *ace* | Pearson Correlation | 0.142 | 0.871** | 0.017 | -0.118 | 0.402** | -0.147 | 0.281 | 1 | |
| | Sig. (2-tailed) | 0.358 | 0 | 0.912 | 0.444 | 0.007 | 0.342 | 0.064 | | . |
| *cyl* | Pearson Correlation | .a | .a | .a | .a | .a | .a | .a | .a | .a |
| | Sig. (2-tailed) | . | . | . | . | . | . | . | . | |

**Correlation is significant at the 0.01 level (2-tailed), .aCan-t be computed because at least one of the variables is constant.

**Supplementary Table S2.** Association in the detection of virulence genes and determination of biofilm formation in *E. faecalis* (n = 44) isolated from rectal swab samples of rhesus macaques.

| Virulence genes | Virulence in different degrees of biofilm formation | | | *p*-value |
|---|---|---|---|---|
| | **No. (%) strong biofilm former (n = 9)** | **No. (%) intermediate biofilm former (n = 26)** | **No. (%) non-biofilm former (n = 9)** | |
| *agg* | 9 (100$^{a,b}$) | 26 (100$^{b}$) | 7 (77.8$^{a}$) | 0.017 |
| *fsrA* | 9 (100$^{a}$) | 26 (100$^{a}$) | 0 (0$^{b}$) | <0.001 |
| *fsrB* | 9 (100$^{a}$) | 23 (88.5$^{a}$) | 9 (100$^{a}$) | 0.328 |
| *fsrC* | 9 (100$^{a}$) | 25 (96.2$^{a}$) | 9 (100$^{a}$) | 0.702 |
| *pil* | 9 (100$^{a,b}$) | 26 (100$^{b}$) | 7 (77.8$^{a}$) | 0.017 |
| *gelE* | 9 (100$^{a}$) | 23 (88.5$^{a}$) | 9 (100$^{a}$) | 0.328 |
| *sprE* | 9 (100$^{a}$) | 26 (100$^{a}$) | 8 (88.9$^{a}$) | 0.137 |
| *ace* | 9 (100$^{a}$) | 26 (100$^{a}$) | 0 (0$^{b}$) | <0.001 |
| *cyl* | 0 (0$^{a}$) | 0 (0$^{a}$) | 0 (0$^{a}$) | NA |

Here, values with different superscripts differ significantly ($P < 0.05$) within the variable under assessment, CI= Confidence interval, NA= Not applied.

**Supplementary Table S3.** Association of antibiotic resistance patterns and biofilm formation in *E. faecalis* strains detected in rectal swab samples of rhesus macaques.

| Categories | Antibiotics | Antibiotic resistance in different degrees of biofilm formation | | | *p*-value |
|---|---|---|---|---|---|
| | | **No. (%) strong biofilm former (n = 9)** | **No. (%) intermediate biofilm former (n = 26)** | **No. (%) non-biofilm former (n = 9)** | |
| **Phenotypic** | **CIP** | 0 (0$^{a}$) | 0 (0$^{a}$) | 0 (0$^{a}$) | NA |
| | **TE** | 2 (22.2$^{a}$) | 6 (23.1$^{a}$) | 1 (11.1$^{a}$) | 0.737 |
| | **LEV** | 0 (0$^{a}$) | 0 (0$^{a}$) | 0 (0$^{a}$) | NA |
| | **FOS** | 0 (0$^{a}$) | 0 (0$^{a}$) | 0 (0$^{a}$) | NA |
| | **RD** | 9 (100$^{a}$) | 26 (100$^{a}$) | 9 (100$^{a}$) | NA |
| | **P** | 9 (100$^{a}$) | 26 (100$^{a}$) | 9 (100$^{a}$) | NA |
| | **LZD** | 9 (100$^{a}$) | 16 (61.5$^{a}$) | 1 (11.1$^{b}$) | 0.001 |
| | **NOR** | 0 (0$^{a}$) | 0 (0$^{a}$) | 0 (0$^{a}$) | NA |
| | **NIT** | 0 (0$^{a}$) | 0 (0$^{a}$) | 0 (0$^{a}$) | NA |
| | **AMP** | 9 (100$^{a}$) | 26 (100$^{a}$) | 9 (100$^{a}$) | NA |
| | **C** | 2 (22.2$^{a}$) | 1 (3.8$^{a}$) | 0 (0$^{a}$) | 0.112 |
| | **VA** | 9 (100$^{a}$) | 13 (50$^{b}$) | 1 (11.1$^{b}$) | 0.001 |
| | **E** | 9 (100$^{a}$) | 15 (57.7$^{a,b}$) | 2 (22.2$^{b}$) | 0.003 |
| **Genotypic** | ***bla*$_{TEM}$** | 9 (100$^{a}$) | 12 (46.2$^{b}$) | 6 (66.7$^{a,b}$) | 0.016 |

Here, values with different superscripts differ significantly ($P < 0.05$) within the variable under assessment, CI= Confidence interval, NA= Not applied.

**Supplementary Table S4:** Pearson correlation coefficient to assess the pairs of any of two virulence genes detected in *E. faecium* isolated from rectal swab samples of rhesus macaques

| **Correlations** | | | | | | | | | | |
|---|---|---|---|---|---|---|---|---|---|---|
| | | ***agg*** | ***fsrA*** | ***fsrB*** | ***fsrC*** | ***pil*** | ***gelE*** | ***sprE*** | ***ace*** | ***cyl*** |
| *agg* | Pearson Correlation | 1 | | | | | | | | |
| | Sig. (2-tailed) | | | | | | | | | |
| *fsrA* | Pearson Correlation | 0.48 | 1 | | | | | | | |
| | Sig. (2-tailed) | 0.07 | | | | | | | | |
| *fsrB* | Pearson Correlation | 0.48 | 0.722** | 1 | | | | | | |
| | Sig. (2-tailed) | 0.07 | 0.002 | | | | | | | |
| *fsrC* | Pearson Correlation | 0.48 | 0.722** | 0.722** | 1 | | | | | |
| | Sig. (2-tailed) | 0.07 | 0.002 | 0.002 | | | | | | |
| *pil* | Pearson Correlation | 0.207 | -0.185 | -0.185 | -0.185 | 1 | | | | |
| | Sig. (2-tailed) | 0.459 | 0.51 | 0.51 | 0.51 | | | | | |
| *gelE* | Pearson Correlation | 0.48 | 0.722** | 0.722** | 0.722** | -0.185 | 1 | | | |
| | Sig. (2-tailed) | 0.07 | 0.002 | 0.002 | 0.002 | 0.51 | | | | |
| *sprE* | Pearson Correlation | 0.48 | 0.444 | 0.444 | 0.444 | 0.431 | 0.444 | 1 | | |
| | Sig. (2-tailed) | 0.07 | 0.097 | 0.097 | 0.097 | 0.109 | 0.097 | | | |
| *ace* | Pearson Correlation | 0.650** | 0.739** | 0.739** | 0.739** | -0.023 | 0.739** | 0.739** | 1 | |
| | Sig. (2-tailed) | 0.009 | 0.002 | 0.002 | 0.002 | 0.936 | 0.002 | 0.002 | | |
| *cyl* | Pearson Correlation | 0.423 | 0.48 | 0.48 | 0.48 | -0.237 | 0.48 | 0.48 | 0.650** | 1 |
| | Sig. (2-tailed) | 0.116 | 0.07 | 0.07 | 0.07 | 0.396 | 0.07 | 0.07 | 0.009 | |

**Correlation is significant at the 0.01 level (2-tailed).

**Supplementary Table S05.** Association in the detection of virulence genes and determination of biofilm formation in *E. faecium* (n = 15) isolated from rectal swab samples of rhesus macaques.

| **Virulence genes** | **Virulence in different degrees of biofilm formation** | | | ***p*-value** |
|---|---|---|---|---|
| | **No. (%) strong biofilm former (n = 3)** | **No. (%) intermediate biofilm former (n = 9)** | **No. (%) non-biofilm former (n = 3)** | |
| *agg* | 2 (66.7[a]) | 0 (0[b]) | 0 (0[a,b]) | 0.010 |
| *fsrA* | 3 (100[a]) | 3 (33.3[a,b]) | 0 (0[b]) | 0.036 |
| *fsrB* | 3 (100[a]) | 3 (33.3[a,b]) | 0 (0[b]) | 0.036 |
| *fsrC* | 3 (100[a]) | 3 (33.3[a,b]) | 0 (0[b]) | 0.036 |
| *pil* | 1 (33.3[a]) | 3 (33.3[a]) | 0 (0[a]) | 0.506 |
| *gelE* | 3 (100[a]) | 3 (33.3[a,b]) | 0 (0[b]) | 0.036 |
| *sprE* | 3 (100[a]) | 3 (33.3[a,b]) | 0 (0[b]) | 0.036 |
| *ace* | 3 (100[a]) | 1 (11.1[b]) | 0 (0[b]) | 0.005 |
| *cyl* | 0 (0[a]) | 0 (0[a]) | 0 (0[a]) | NA |

Here, values with different superscripts differ significantly ($P < 0.05$) within the variable under assessment, CI= Confidence interval, NA= Not applied.

**Supplementary Table S6.** Association of antibiotic resistance patterns and biofilm formation in *E. faecium* strains detected in rectal swab samples of rhesus macaques.

| **Categories** | **Antibiotics** | **Antibiotic resistance in different degrees of biofilm formation** | | | ***p*-value** |
|---|---|---|---|---|---|
| | | **No. (%) strong biofilm former (n = 3)** | **No. (%) intermediate biofilm former (n = 9)** | **No. (%) non-biofilm former (n = 3)** | |
| **Phenotypic** | **CIP** | 0 (0$^{a}$) | 0 (0$^{a}$) | 0 (0$^{a}$) | NA |
| | **TE** | 0 (0$^{a}$) | 0 (0$^{a}$) | 0 (0$^{a}$) | NA |
| | **LEV** | 0 (0$^{a}$) | 0 (0$^{a}$) | 0 (0$^{a}$) | NA |
| | **FOS** | 0 (0$^{a}$) | 0 (0$^{a}$) | 0 (0$^{a}$) | NA |
| | **RD** | 3 (100$^{a}$) | 9 (100$^{a}$) | 3 (100$^{a}$) | NA |
| | **P** | 3 (100$^{a}$) | 9 (100$^{a}$) | 3 (100$^{a}$) | NA |
| | **LZD** | 3 (100$^{a}$) | 6 (66.7$^{a,b}$) | 0 (0$^{b}$) | 0.036 |
| | **NOR** | 0 (0$^{a}$) | 0 (0$^{a}$) | 0 (0$^{a}$) | NA |
| | **NIT** | 0 (0$^{a}$) | 0 (0$^{a}$) | 0 (0$^{a}$) | NA |
| | **AMP** | 3 (100$^{a}$) | 9 (100$^{a}$) | 3 (100$^{a}$) | NA |
| | **C** | 0 (0$^{a}$) | 0 (0$^{a}$) | 0 (0$^{a}$) | NA |
| | **VA** | 3 (100$^{a}$) | 4 (44.4$^{a,b}$) | 0 (0$^{b}$) | 0.048 |
| | **E** | 3 (100$^{a}$) | 9 (100$^{a}$) | 3 (100$^{a}$) | NA |
| **Genotypic** | $\boldsymbol{bla}_{TEM}$ | 3 (100$^{a}$) | 6 (66.7$^{a,b}$) | 0 (0$^{b}$) | 0.036 |

Here, values with different superscripts differ significantly ($P < 0.05$) within the variable under assessment, CI = Confidence interval, NA= Not applied.
